# Chemokine Ligand 2 Promotes Migration in Osteosarcoma by Regulating the miR-3659/MMP-3 Axis

**DOI:** 10.3390/biomedicines11102768

**Published:** 2023-10-12

**Authors:** Yu-Hsiang Chang, Yuan-Li Huang, Hsiao-Chi Tsai, An-Chen Chang, Chih-Yuan Ko, Yi-Chin Fong, Chih-Hsin Tang

**Affiliations:** 1 Program for Cancer Biology and Drug Discovery, China Medical University, Taichung 404328, Taiwan; billy0225@icloud.com; 2 Department of Medical Laboratory Science and Biotechnology, Asia University, Taichung 41354, Taiwan; yuanli@asia.edu.tw; 3 Department of Medical Education and Research, China Medical University Beigang Hospital, Yunlin 651012, Taiwan; moxa0110@gmail.com; 4 Division of Hematology and Oncology, Department of Internal Medicine, China Medical University Hospital, Taichung 404327, Taiwan; 5 Translational Medicine Center, Shin-Kong Wu Ho-Su Memorial Hospital, Taipei 111045, Taiwan; annone3212@gmail.com; 6 Graduate Institute of Biomedical Sciences, China Medical University, Taichung 404328, Taiwan; d14333@mail.cmuh.org.tw; 7 Department of Orthopedic Surgery, China Medical University Hospital, Taichung 404327, Taiwan; 8 Department of Sports Medicine, College of Health Care, China Medical University, Taichung 404328, Taiwan; 9 Department of Orthopedic Surgery, China Medical University Beigang Hospital, Yunlin 651012, Taiwan; 10 Department of Pharmacology, School of Medicine, China Medical University, Taichung 404328, Taiwan; 11 Chinese Medicine Research Center, China Medical University, Taichung 404328, Taiwan; 12 Department of Medical Research, China Medical University Hsinchu Hospital, Hsinchu 302056, Taiwan

**Keywords:** osteosarcoma, CCL2, MMP-3, metastasis, miR-3659

## Abstract

Osteosarcoma is a common malignant tumor in children and adolescents, known for its aggressive invasion and distant metastasis, leading to a poor prognosis. Matrix metalloproteinases (MMPs) can degrade the extracellular matrix and basement membranes through their proteolytic activity, thereby promoting osteosarcoma metastasis. Chemokine ligand 2 (CCL2) is a well-studied chemokine that plays a significant role in the cell motility of many cancers. However, its specific involvement in osteosarcoma metastasis is not fully understood. The aim of this study is to examine the role of miRNAs in CCL2-mediated MMP expression and cell motility in human osteosarcoma. The analysis of immunohistochemistry data and databases associated a positive correlation between CCL2 or MMP-3 levels with the metastasis of osteosarcoma patients. The in vivo lung metastatic osteosarcoma model also demonstrated similar effects, showing higher levels of CCL2 and MMP-3 in lung metastatic osteosarcoma tissues. The stimulation of osteosarcoma cells with CCL2 enhanced migration and invasion abilities through the upregulation of MMP-3 synthesis. Our results also indicate that CCL2 enhances MMP-3-dependent cell motility by inhibiting miR-3659 synthesis. Therefore, CCL2 represents a promising therapeutic target for treating metastasis in osteosarcoma.

## 1. Introduction

Osteosarcoma is a mesenchymal malignancy characterized by the formation of immature bone by tumor cells [1]. It most commonly occurs in adolescents but also has a second peak in incidence among the elderly [2]. The main treatment methods for osteosarcoma include surgical resection of the primary tumor and systemic chemotherapy [3]. However, treatment strategies have not significantly improved the prognosis for osteosarcoma patients over the past forty years [4]. Previous studies have shown that osteosarcoma is highly aggressive, with 20% of patients exhibiting evident metastases to distant organs at initial diagnosis [5]. Additionally, patients with osteosarcoma without lung metastases have a 5-year survival rate of 70%, while those with lung metastases have a survival rate of 30% [6]. There is an alarming recurrence rate of 80% among these 30% of osteosarcoma cases [7]. Therefore, the difficulty in treating osteosarcoma stems from its tendency to metastasize.

Metastasis is a complex process involving changes in cell adhesion properties, motility, and most importantly, the degradation of the extracellular matrix (ECM) [8]. Consequently, cancer cells escape from the primary lesion and gradually spread to distant organs through migration and invasion [8,9]. The ECM is composed of various biochemically distinct proteins and is involved in a variety of biological processes [10], such as differentiation, adhesion, migration, and communication. It provides necessary structural and biochemical support for its cellular constituents [4]. According to the report, osteosarcoma is characterized by its abundant pathological osteoid ECM [11]. This implies that the abnormal signaling and structural components of the ECM are the main drivers of osteosarcoma development, playing a critical role in the progression and metastasis of osteosarcoma [4].

Based on previous research, integrins, cell adhesion molecules (CAMs), and matrix metalloproteinases (MMPs) are implicated in several critical processes, including tumor metastasis [12]. The degradation of the ECM is primarily achieved through proteases [9], such as MMPs, which are the major enzymes involved in ECM degradation [10]. Studies have shown that MMP-9 can facilitate ECM degradation, allowing osteosarcoma cells to penetrate the ECM and metastasize to other tissues and organs [13,14]. It has been found that MMP-3 is more highly expressed in metastatic osteosarcoma than in primary osteosarcoma tumor tissues [15]. Additionally, the downregulation of MMP-3 and MMP-13 can decrease osteosarcoma cell migration and invasion [15,16,17]. It has been reported that *MMP3* is modulated by several microRNAs (miRNAs and miRs), which regulate cell motility, metastasis, and tumor prognosis [18,19]. Therefore, researching the activation mechanism of MMPs and the involvement of miRNAs will help us understand the processes of cellular motility, invasion, and migration in osteosarcoma metastasis.

Previous research has indicated that MMPs are regulated by various cytokines [20]. For example, chemokine ligand 2 (CCL2) is one of the cytokines involved in the regulation of MMPs [21,22,23]. CCL2 is the first widely studied C-C chemokine [24] and it plays a critical role in recruiting monocytes and macrophages to inflammatory sites while also regulating their activity [25]. Importantly, it has been reported that CCL2 can promote the development and progression of many types of cancers by directly and indirectly stimulating cancer cell migration through the regulation of MMPs [21,22]. However, the effect of CCL2 on MMP regulation in the cell motility of osteosarcoma remains largely uninvestigated. The aim of this study is to examine the role of miRNAs in CCL2-mediated MMP expression and cell motility in human osteosarcoma. We found higher levels of CCL2 expression in human metastatic osteosarcoma samples with non-metastatic osteosarcoma or normal healthy bone samples. We indicated that CCL2 promotes MMP-3-mediated migration of osteosarcoma. The inhibition of miR-3659 synthesis is involved in CCL2-promoted upregulation of MMP-3 production and migration. These findings suggest that CCL2 could be a valuable target in the context of metastatic osteosarcoma.

## 2. Materials and Methods

### 2.1. Cell Culture

The human osteosarcoma cell line 143B was bought from the Bioresource Collection and Research Center (BCRC) in Hsinchu, Taiwan. The 143B cells were maintained in Dulbecco’s Modified Eagle Medium (DMEM) from Grand Island, NY, USA, and supplemented with penicillin (100 U/mL), streptomycin (100 μg/mL), and 10% FBS (Grand Island, NY, USA), and cultured at 37 °C with 5% CO_2_.

### 2.2. Analysis of mRNA Expression Profiles from the Cancer Genome Atlas (TCGA) Database

The transcriptome profiles of osteosarcoma in the TCGA database were downloaded using UCSC Xena (http://xena.ucsc.edu, accessed on 17 October 2022). A total of 88 osteosarcoma samples that had been analyzed through RNA sequencing were used to study the gene expression profiles of *CCL2* and *MMP3* [26].

### 2.3. Immunohistochemistry (IHC) Staining

The tissue slides of osteosarcoma were baked at 60 °C overnight and then deparaffinized using xylene. They were subsequently rehydrated through an ethanol series. To block endogenous peroxidase activity, 3% hydrogen peroxide in methanol was applied for 10 min. Heat-induced antigen retrieval was performed for all sections using 0.01 M sodium citrate buffer at pH 6, heated to 95 °C for 25 min. The rabbit monoclonal antibody for CCL2 (Abcam, Cambridge, MA, USA) and the mouse monoclonal antibody for MMP-3 (Santa Cruz Biotechnology, Santa Cruz, CA, USA) were diluted 1:200 and left to incubate overnight at 4 °C. The antibody-binding signal was detected using the NovoLink Polymer Detection System (Leica Microsystems, Wetzlar, Germany) and visualized using the diaminobenzidine reaction. The sections were counterstained with hematoxylin. The immunostaining intensity of CCL2 and MMP-3 was scored using the MacBiophotonics Image J software (version 1.53) [27].

### 2.4. Cell Migration and Invasion Assay

Osteosarcoma cells were applied into the upper chamber of transwell plates (Costar, NY, USA) precoated with a layer of Matrigel for the invasion assay. Cells were stimulated with CCL2 (PeproTech, Rocky Hill, NJ, USA) to the lower chamber. After 24 h, the migrated cells were fixed with 3.7% formaldehyde and stained with crystal violet, then quantified under the microscope [28].

### 2.5. Quantitative Real-Time PCR (q-PCR)

Total RNA was extracted from osteosarcoma cells using TRIzol reagent (MDBio Inc., Taipei, Taiwan). The reverse transcription reaction was performed using 2 μg of total RNA, which was reverse transcribed into complementary DNA (cDNA) using an oligo (dT) primer. Then, 100 ng of total cDNA was mixed with sequence-specific primers using a KAPA SYBR^®^ FAST qPCR Kit (Applied Biosystems, Foster City, CA, USA). The specific forward primer sequence for *GAPDH* is 5′-ACCACAGTCCATGCCATCAC-3′ and the reverse primer sequence for *GAPDH* is 5′-TCCACCACCCTGTTGCTGTA-3′. The specific forward primer sequence for *MMP3* is 5′-CTGGACTCCGACACTCTGGA-3′ and the specific reverse primer sequence for *MMP3* is 5′-CAGGAAAGGTTCTGAAGTGACC-3′. RT-qPCR assays were performed in triplicate using a StepOnePlus sequence detection system. The cycling conditions consisted of 10 min of polymerase activation at 95 °C followed by 40 cycles at 95 °C for 15 s and 60 °C for 60 s. The levels of *GAPDH* expression were used as an endogenous control for normalization purposes. Results were expressed as Ct values and normalized to calculate the average Ct of each sample (ΔCt). For the detection of miRNAs, reverse transcription was performed with the Mir-XTM miRNA First-Strand Synthesis and SYBR^®^ qRT-PCR (Clontech Laboratories, Inc., Mountain View, CA, USA). The specific primer sequence for miR-4641 is 5′-TGCCCATGCCATACTTTTGCCTCA-3′, miR-17-5p is 5′-CAAAGTGCTTACAGTGCAGGTAG-3′, miR-106a-5p is 5′-AAAAGTGCTTACAGTGCAGGTAG-3′, miR-3659 is 5′-TGAGTGTTGTCTACGAGGGCA-3′, miR-574-3p is 5′-CACGCTCATGCACACACCCACA-3′, miR-5683 is 5′-TACAGATGCAGATTCTCTGACTTC-3′, miR-31-5p is 5′-AGGCAAGATGCTGGCATAGCT-3′, miR-4690-3p is 5′-GCAGCCCAGCTGAGGCCTCTG-3′, miR-18a-5p is 5′-TAAGGTGCATCTAGTGCAGATAG-3′, miR-18b-5p is 5′-TAAGGTGCATCTAGTGCAGTTAG-3′, miR-3153 is 5′-GGGGAAAGCGAGTAGGGACATTT-3′, and miR-1231 is 5′-GTGTCTGGGCGGACAGCTGC-3′. An expression level of U6 was used for normalization [29,30].

### 2.6. Analysis of Clinical Samples

Human bone and tumor tissues were obtained during primary total knee arthroplasty and from patients diagnosed with osteosarcoma undergoing orthopedic surgery at China Medical University Hospital, with approval granted by the local Institutional Review Board. All study participants gave written consent before enrollment.

### 2.7. Cell Transfection

MMP-3 or control siRNAs were purchased commercially from Santa Cruz Biotechnology (Santa Cruz, CA, USA). MiR-3659 mimic or control mimic were purchased commercially from AllBio Science (Taipei, Taiwan). The specific sequence for miR-3659 mimic is 5′-UGAGUGUUGUCUACGAGGGCA-3′ and control mimic is 5′-UUGUACUACACAAAAGUACUG-3′. Transient transfection was conducted using Lipofectamine 2000 (Invitrogen Life Technology, Carlsbad, CA, USA) according to the manufacturer’s instructions.

### 2.8. Western Blot

Cell lysates were resolved by SDS-PAGE and transferred to Immobilon polyvinylidene fluoride membranes. Blots were blocked with skim milk for 1 h at room temperature, then probed with MMP-3 (1:500) (Santa Cruz Biotechnology, Santa Cruz, CA, USA) or β-actin antibodies (1:10,000) (Merck, Darmstadt, Germany) for 1 h at room temperature. After undergoing three washes, blots were incubated with peroxidase-conjugated secondary antibody (1:2000) for another hour at room temperature. The blots were developed via enhanced chemiluminescence and visualized using the iBright^TM^ CL1500 Imaging System (Waltham, MA, USA).

### 2.9. Statistics

All statistical data were analyzed using GraphPad Prism 9.0 (GraphPad Software, San Diego, CA, USA) and are presented as the mean ± standard deviation (S.D.). Statistical comparisons between two samples were performed using Student’s *t*-test. One-way analysis of variance (ANOVA) with post hoc Bonferroni correction was conducted for statistical analysis of multiple groups. In all cases, a *p*-value of <0.05 was considered significant.

## 3. Results

### 3.1. Elevated Levels of CCL2 Expression in Patients with Metastatic Osteosarcoma

**Figure 1:**
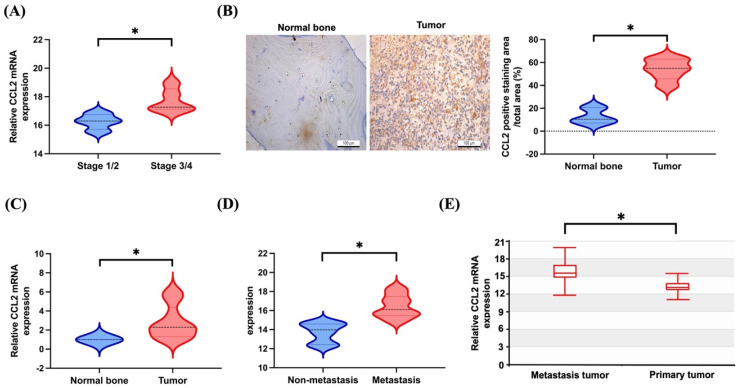
High levels of CCL2 expression in patients with osteosarcoma metastatic disease. (**A**,**D**) Levels of *CCL2* expression in osteosarcoma samples retrieved from TCGA dataset. (**B**) Representative images of IHC staining for CCL2 in tissue samples from healthy individuals and osteosarcoma patients are depicted. (**C**) The mRNA expression of *CCL2* in the indicated group was examined by qPCR. (**E**) *CCL2* expression levels in metastatic and primary osteosarcoma retrieved from the Human Cancer Metastasis Database. * *p* < 0.05 compared to control.

The TCGA database indicated that patients with higher stages (stage 3/4) of osteosarcoma exhibited significantly elevated levels of *CCL2* expression compared to those observed in lower-stage patients (stage 1/2) (Figure 1A). Our clinical data also found that CCL2 protein and mRNA expression levels in osteosarcoma patients were significantly higher than in normal individuals (Figure 1B,C). Records from the TCGA database and the Human Cancer Metastasis database revealed markedly elevated *CCL2* expression levels in metastatic osteosarcoma samples, surpassing those in primary osteosarcoma samples (Figure 1D,E). Our findings underscore the correlation between heightened CCL2 levels and adverse outcomes, including cancer metastasis, in osteosarcoma.

### 3.2. CCL2 Increases Cell Migration and Invasion in Osteosarcoma by Enhancing MMP-3 Production

**Figure 2:**
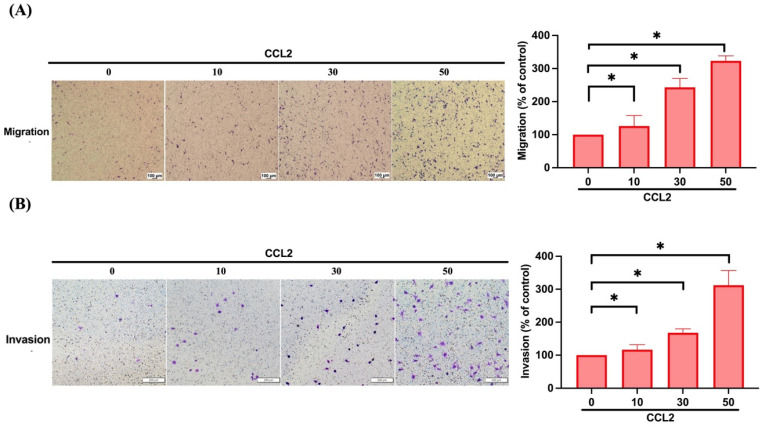
CCL2 promotes cell migration and invasion in human osteosarcomas. (**A**,**B**) The 143B cells were treated with various concentrations of CCL2 (0–50 ng/mL) and cell migration and invasion abilities were assessed using the transwell assay. * *p* < 0.05 compared to control.

**Figure 3:**
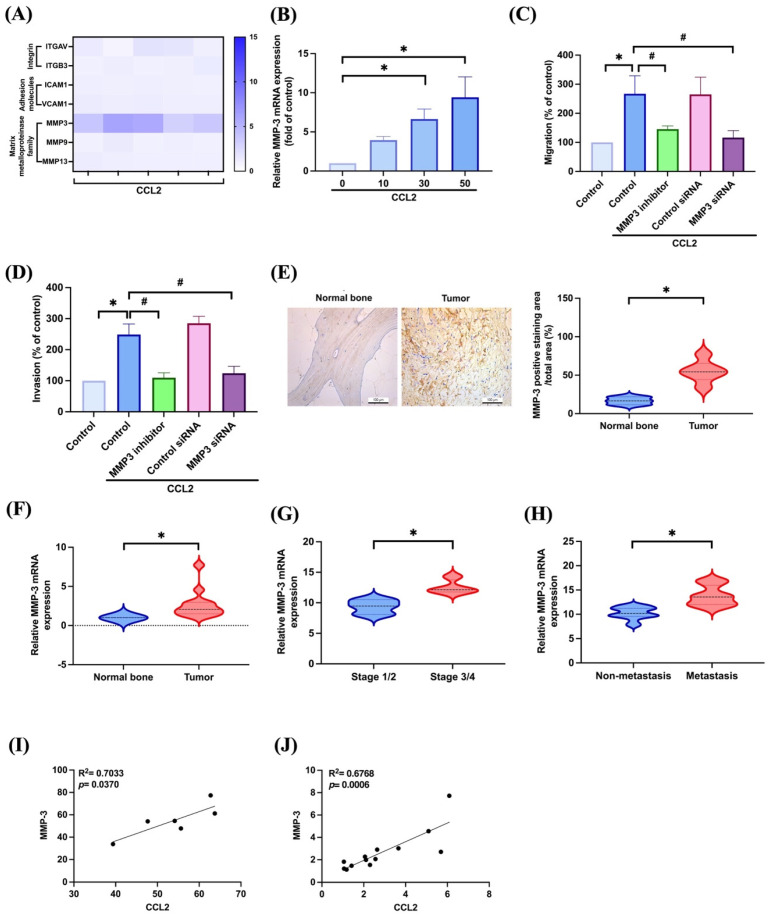
MMP-3 is involved in CCL2-promoted cell migration and invasion in osteosarcomas. (**A**) Cells were treated with CCL2 (50 ng/mL) for 24 h and the indicated mRNA expression was detected by qPCR. (**B**) Cells were treated with CCL2 (0–50 ng/mL) for 24 h and the *MMP3* expression was examined by qPCR. (**C**,**D**) Cells were treated with an MMP-3 inhibitor (10 μM) or siRNA and then stimulated with CCL2 (50 ng/mL); the cell migration and invasion were examined. (**E**,**F**) IHC staining and qPCR for MMP-3 expression in healthy individuals and osteosarcoma patients are presented. (**G**,**H**) The level of *MMP3* expression in indicated osteosarcoma tissues recovered from the TCGA dataset. (**I**,**J**) Correlation of CCL2 and MMP-3 protein and mRNA levels in clinical osteosarcoma tissues. * *p* < 0.05 compared to control. # *p* < 0.05 compared to CCL2-treated group.

Next, we directly employed the transwell assay, a well-established model for examining cell motility following CCL2 treatment in osteosarcoma cells. Our findings revealed a concentration-dependent effect of CCL2 on osteosarcoma cell migration (Figure 2A). Furthermore, CCL2 increased cell invasiveness through Matrigel (Figure 2B). Consequently, CCL2 promoted both migration and invasion in osteosarcoma. Our results revealed a robust increase in *MMP3* mRNA expression after CCL2 stimulation in osteosarcoma cells compared to others (Figure 3A). Moreover, the expression of *MMP3* mRNA demonstrated a concentration-dependent response upon CCL2 induction (Figure 3B). To confirm MMP-3’s involvement in osteosarcoma cell migration and invasion driven by CCL2, we treated osteosarcoma cells with an MMP-3 inhibitor or siRNA, both of which resulted in the inhibition of CCL2-induced enhancement of cell migration and invasion (Figure 3C,D). Subsequently, we conducted IHC staining to observe MMP-3 levels in osteosarcoma patients. Our clinical sample results showed significantly higher MMP-3 protein and mRNA expression in osteosarcoma patients than in normal individuals (Figure 3E,F). *MMP3* expression levels in stage 3/4 and metastatic osteosarcoma samples are higher than those in stage 1/2 and primary osteosarcoma samples, respectively (Figure 3G,H). Finally, we analyzed the correlation between CCL2 and MMP-3 and the results indicated a positive correlation between them (Figure 3I,J). Thus, our findings suggest that MMP-3 is indeed involved in CCL2-induced osteosarcoma cell migration and invasion.

### 3.3. Inhibiting miR-3659 Regulates CCL2-Induced Promotion of MMP-3 Production and Motility in Osteosarcoma

**Figure 4:**
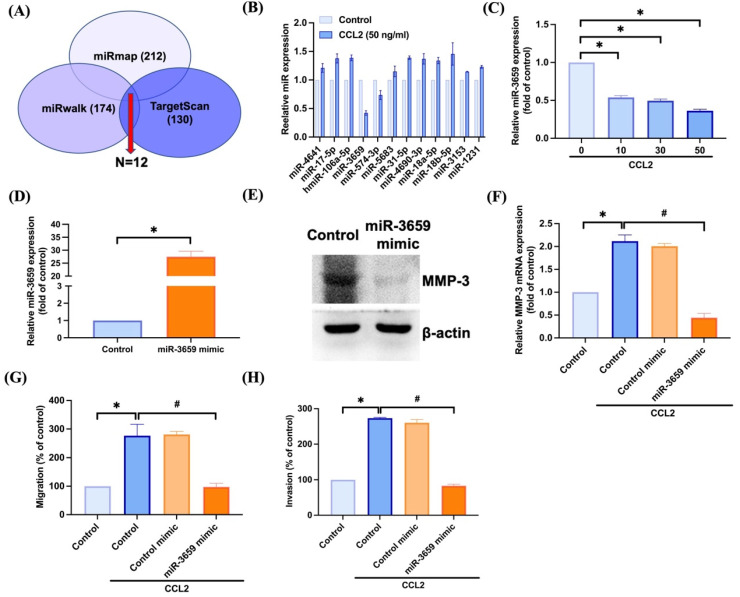
Inhibition miR-3659 regulates CCL2-mediated MMP-3 generation and cell motility. (**A**) The miRmap, miRwalk, and TargetScan computational software were used to identify potential miRNAs that bind to *MMP3*. (**B**,**C**) Cells were treated with CCL2 for 24 h and miRNA’s expression was examined by qPCR. (**D**,**E**) Cells were transfected with miRNA mimic (20 nM) and the mRNA expression of miR-3659 and protein expression of MMP-3 were examined by qPCR and western blot. (**F**–**H**) Cells were transfected with miRNA control (20 nM) or miR-3659 mimic (20 nM) and then stimulated with CCL2 (50 ng/mL); the *MMP3* expression, cell migration, and invasion were examined. * *p* < 0.05 compared to control. # *p* < 0.05 compared to CCL2-treated group.

To further analyze potential miRNAs regulating *MMP3*, we initially utilized open-source software (miRWalk, TargetScan, and miRmap) to determine the miRNAs that regulate *MMP3* expression. Among these databases, miRmap contained 212 miRNAs, miRWalk contained 174 miRNAs, and TargetScan contained 130 miRNAs that were predicted to bind to *MMP3* mRNA (Figure 4A). Upon intersecting these sets, we found that 12 miRNAs were involved (Figure 4A). The qPCR results indicated that miR-3659 was significantly downregulated compared to the others after CCL2 stimulation (Figure 4B). Treatment with CCL2 led to a concentration-dependent reduction in miR-3659 expression (Figure 4C). To investigate whether miR-3659 mediates CCL2-induced MMP-3-mediated cell migration, we employed a miR-3659 mimic. Transfection with the miR-3659 mimic enhanced the expression of miR-3659 and inhibited the protein level of MMP-3 (Figure 4D,E). The miR-3659 mimic also counteracted CCL2-induced facilitation of *MMP3* production, cell migration, and invasion (Figure 4F–H). Therefore, miR-3659 plays a crucial role in CCL2-induced osteosarcoma cell motility by regulating MMP-3 expression.

### 3.4. CCL2 and MMP-3 Are Highly Expressed in Lung Metastatic Osteosarcoma In Vivo

**Figure 5:**
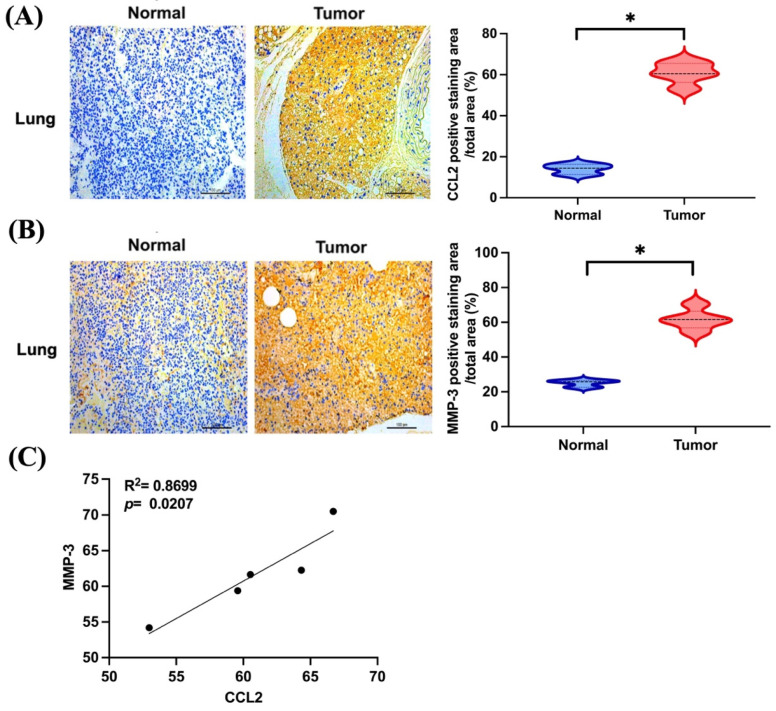
Higher expression of CCL2 and MMP-3 in lung metastatic osteosarcoma in vivo. (**A**,**B**) Representative images of IHC staining for CCL2 and MMP-3 in lung tissues for normal and metastatic osteosarcoma cases are presented. (**C**) The correlation of CCL2 and MMP-3 in metastatic osteosarcoma tissues. * *p* < 0.05 compared to control.

Next, we further examined the expression levels of CCL2 and MMP-3 in metastatic osteosarcoma tissues in vivo from our previous study [7]. Both CCL2 and MMP-3 were highly expressed in lung metastatic osteosarcoma tissues compared to normal lung tissues (Figure 5A,B). The correlation between CCL2 and MMP-3 was found to be positive in osteosarcoma (Figure 5C). Therefore, CCL2 and MMP-3 are highly expressed in metastatic osteosarcoma in vivo.

## 4. Discussion

CCL2 is a chemokine that can activate NK cells, memory T lymphocytes, macrophages, and monocytes, stimulating the release of proinflammatory cytokines including TNF-α and IL-6 [24,31]. Conversely, CCL2-activated macrophages also secrete tissue repair factors, such as TGF-β and VEGF [31]. Numerous studies have shown that the overexpression of CCL2 amplifies many cancer cells’ motility and promotes tumor development and metastasis [32,33,34,35,36,37,38], such as chondrosarcoma [22], nasopharyngeal carcinoma [21], and breast carcinoma [23]. However, there is limited research on the role of CCL2 in osteosarcoma metastasis. Therefore, this study primarily focuses on investigating the ability of CCL2 to facilitate metastasis in osteosarcoma. In this study, we first examined the *CCL2* levels in patients with osteosarcoma through an analysis of the TCGA database and the Human Cancer Metastasis Database and we confirmed the significant correlation between *CCL2* expression and osteosarcoma stage, as well as distance metastasis. We further investigated the impact of CCL2 on the migratory and invasive capacities of osteosarcoma cells in vitro and in vivo. These findings further emphasize the importance of CCL2 in osteosarcoma and suggest its potential as a therapeutic target.

Osteosarcoma, a rare but highly aggressive malignancy in children and adolescents [39,40,41,42], is characterized by its ability to metastasize, leading to treatment failure and a poor prognosis [43,44]. Therefore, metastases in osteosarcoma are the primary cause of death in patients [19]. The metastatic cascade involves invading cells by altering intracellular adhesion properties, reorganizing the ECM environment, and restructuring their cell skeletons. As a result, tumor cells escape from the primary lesion and gradually spread to distant organs. Accumulating reports indicate that CAMs play a critical role in modulating cell-matrix adhesions [8,45,46]. For example, an overexpressed intercellular adhesion molecule-1 (ICAM-1) can promote osteosarcoma metastasis [8]. Upregulating the expression of the vascular cell adhesion molecule 1 (VCAM-1) stimulates human osteosarcoma cell migration and drives osteosarcoma lung metastasis [45,46]. Additionally, integrins, major receptors for cell adhesion to ECM molecules, modulate cell migration and tissue remodeling [47]. Integrin αvβ3 may regulate the interaction between osteosarcoma cells and the ECM, thereby promoting osteosarcoma cell migration [48,49]. Moreover, MMPs, zinc-dependent endopeptidases, have proteolytic activity that helps degrade ECM and basement membranes [19,50,51]. For example, MMP-9 facilitates ECM degradation, enabling osteosarcoma cells to penetrate the ECM and metastasize to other tissues and organs [13]. The downregulation of MMP-13 is also identified as a crucial mechanism decreasing osteosarcoma cell migration and invasion [16].

The inhibition of MMP-3 decreases osteosarcoma cell migration and invasion [15,16,17]. MMP-3 is more highly expressed in metastatic osteosarcoma than in primary osteosarcoma tumor tissues [15]. In this study, our results found that CCL2 strongly upregulated MMP-3 expression compared to other MMPs, CAMs, and integrins. The expression level of MMP-3 in osteosarcoma patients was significantly higher than in the normal group. We previously established an osteosarcoma lung metastasis model by injecting 143B osteosarcoma cells into the lateral tail vein to induce the development of lung metastasis [7]. In this study, we observed higher levels of CCL2 and MMP-3 expression in lung metastatic osteosarcoma tissues compared to normal lung tissues. Importantly, the CCL2-induced promotion of migration and invasion was abolished by treatment with an MMP-3 inhibitor. Additionally, genetic inhibition using siRNA against *MMP3* yielded similar results. These findings establish MMP-3 as a key regulator in the process of tumor metastasis in osteosarcoma induced by CCL2.

*MMP3* is regulated by various miRNAs [18,19], subsequently facilitating tumor cell invasion and metastasis [15,16,17]. The abnormal expression of miRNAs has been closely associated with processes such as tumor cell invasion and metastasis in osteosarcoma [18,19,52,53,54]. For example, the increased expression of MMP-3 induces tumor metastasis in osteosarcoma by inhibiting miR-519d [19], and miR-134 reduces osteosarcoma cell invasion and metastasis by targeting MMP-3 in vivo and in vitro [18]. However, it is not clear which miRNA regulates *MMP3* in CCL2-induced osteosarcoma cell motility. In our research, we utilized open-access prediction software to identify miRNAs that could potentially bind to *MMP3* and subsequently identified miR-3659 as a candidate miRNA that can interact with *MMP3*. The miR-3659 mimic significantly suppressed CCL2-promoted osteosarcoma migration and invasion. These results suggest that CCL2 enhances migration and invasion in osteosarcoma by increasing MMP-3 expression through the inhibition of miR-3659 synthesis.

MiRNAs regulate gene expression through binding to the 3′UTRs of target mRNAs, either targeting the transcripts for degradation or blocking their translation [55]. Here, we did not generate the 3′UTR luciferase plasmids of *MMP3*. Whether miR-3659 directly or indirectly binds to 3′-UTR of *MMP3* upon CCL2 treatment needs further examination. The post-transcriptional regulation of MMPs via miRNAs has been implicated in the regulation of cancer motility [56]. In this study, the transfection of cells with the miR-3659 mimic facilitated the expression of miR-3659. Additionally, transfection with the miR-3659 mimic also diminished the protein expression of MMP-3. Further investigation is required to determine whether post-transcriptional regulation contributes to miR-3659-regulated MMP-3 expression.

## 5. Conclusions

**Figure 6:**
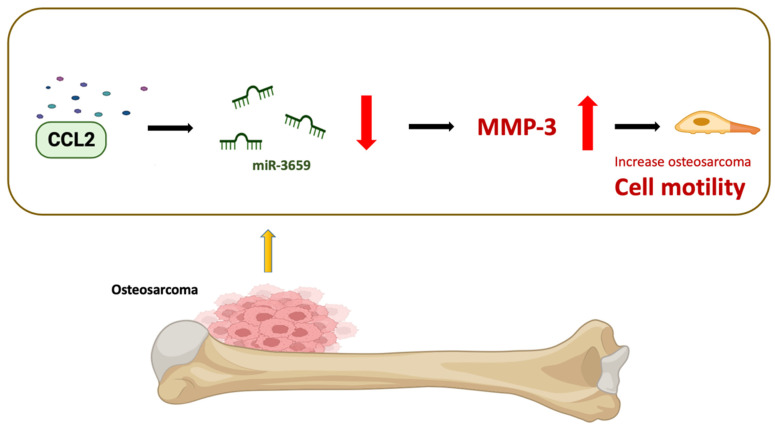
Schematic diagram illustrating the mechanisms of CCL2 function in osteosarcoma metastasis. The schematic sketch summarizes the mechanisms underlying the CCL2-induced increase in MMP-3 production in human osteosarcoma, subsequently enhancing metastasis by inhibiting miR-3659 generation.

In conclusion, our findings have revealed that CCL2 downregulates the expression of miR-3659, consequently increasing the expression of MMP-3, thereby promoting osteosarcoma cell motility (Figure 6). This discovery presents novel targeted options for osteosarcoma treatment, potentially leading to the development of more effective therapeutic strategies and ultimately improving patient prognosis and survival rates.

## Data Availability

The raw data supporting the conclusions of this study are not available.

## References

[1] Belayneh R., Fourman M.S., Bhogal S., Weiss K.R. (2021). Update on osteosarcoma. Curr. Oncol. Rep..

[2] Mirabello L., Troisi R.J., Savage S.A. (2009). Osteosarcoma incidence and survival rates from 1973 to 2004: Data from the surveillance, epidemiology, and end results program. Cancer.

[3] Meltzer P.S., Helman L.J. (2021). New horizons in the treatment of osteosarcoma. N. Engl. J. Med..

[4] Cui J., Dean D., Hornicek F.J., Chen Z., Duan Z. (2020). The role of extracelluar matrix in osteosarcoma progression and metastasis. J. Exp. Clin. Cancer Res..

[5] Zhang J., Li N., Lu S., Chen Y., Shan L., Zhao X., Xu Y. (2021). The role of notch ligand jagged1 in osteosarcoma proliferation, metastasis, and recurrence. J. Orthop. Surg. Res..

[6] Lei T., Qian H., Lei P., Hu Y. (2021). Ferroptosis-related gene signature associates with immunity and predicts prognosis accurately in patients with osteosarcoma. Cancer Sci..

[7] Trang N.T.N., Lai C.Y., Tsai H.C., Huang Y.L., Liu S.C., Tsai C.H., Fong Y.C., Tzeng H.E., Tang C.H. (2023). Apelin promotes osteosarcoma metastasis by upregulating plod2 expression via the hippo signaling pathway and hsa_circ_0000004/mir-1303 axis. Int. J. Biol. Sci..

[8] Hou C.-H., Lin F.-L., Tong K.-B., Hou S.-M., Liu J.-F. (2014). Transforming growth factor alpha promotes osteosarcoma metastasis by icam-1 and pi3k/akt signaling pathway. Biochem. Pharmacol..

[9] Yang J.-S., Lin C.-W., Hsieh Y.-S., Cheng H.-L., Lue K.-H., Yang S.-F., Lu K.-H. (2013). Selaginella tamariscina (beauv.) possesses antimetastatic effects on human osteosarcoma cells by decreasing mmp-2 and mmp-9 secretions via p38 and Akt signaling pathways. Food Chem. Toxicol..

[10] Wang S., Zhong L., Li Y., Xiao D., Zhang R., Liao D., Lv D., Wang X., Wang J., Xie X. (2019). Up-regulation of pcolce by twist1 promotes metastasis in osteosarcoma. Theranostics.

[11] Qin Q., Gomez-Salazar M., Tower R.J., Chang L., Morris C.D., McCarthy E.F., Ting K., Zhang X., James A.W. (2022). nell1 regulates the matrisome to promote osteosarcoma progression. Cancer Res..

[12] Liao Y., Yi Q., He J., Huang D., Xiong J., Sun W., Sun W. (2023). Extracellular vesicles in tumorigenesis, metastasis, chemotherapy resistance and intercellular communication in osteosarcoma. Bioengineered.

[13] Poudel B., Kim D.-K., Ki H.-H., Kwon Y.-B., Lee Y.-M., Kim D.-K. (2014). Downregulation of erk signaling impairs u2os osteosarcoma cell migration in collagen matrix by suppressing mmp9 production. Oncol. Lett..

[14] Liu J.F., Chen P.C., Chang T.M., Hou C.H. (2020). Thrombospondin-2 stimulates mmp-9 production and promotes osteosarcoma metastasis via the plc, pkc, c-src and nf-κb activation. J. Cell Mol. Med..

[15] Huang J.-F., Du W.-X., Chen J.-J. (2016). Elevated expression of matrix metalloproteinase-3 in human osteosarcoma and its association with tumor metastasis. J. BUON.

[16] Deng B., Qiu B. (2015). Shikonin inhibits invasiveness of osteosarcoma through mmp13 suppression. Tumor Biol..

[17] Wang S.-T., Li D.-Z., Li J.-M., Fang J., Li H.-Z., Tong P.-J., Liu F.-C. (2015). Lentivirus-mediated rna interference targeting ubch10 reduces cell growth and invasion of human osteosarcoma cells via inhibition of ki-67 and matrix metalloproteinases. Oncol. Lett..

[18] Chen C.L., Zhang L., Jiao Y.R., Zhou Y., Ge Q.F., Li P.C., Sun X.J., Lv Z. (2019). Mir-134 inhibits osteosarcoma cell invasion and metastasis through targeting mmp1 and mmp3 in vitro and in vivo. FEBS Lett..

[19] Tsai H.-C., Su H.-L., Huang C.-Y., Fong Y.-C., Hsu C.-J., Tang C.-H. (2014). Ctgf increases matrix metalloproteinases expression and subsequently promotes tumor metastasis in human osteosarcoma through down-regulating mir-519d. Oncotarget.

[20] Roomi M.W., Kalinovsky T., Rath M., Niedzwiecki A. (2014). In vitro modulation of mmp-2 and mmp-9 in pediatric human sarcoma cell lines by cytokines, inducers and inhibitors. Int. J. Oncol..

[21] Yang J., Lv X., Chen J., Xie C., Xia W., Jiang C., Zeng T., Ye Y., Ke L., Yu Y. (2016). Ccl2-ccr2 axis promotes metastasis of nasopharyngeal carcinoma by activating erk1/2-mmp2/9 pathway. Oncotarget.

[22] Tang C.-H., Tsai C.-C. (2012). Ccl2 increases mmp-9 expression and cell motility in human chondrosarcoma cells via the ras/raf/mek/erk/nf-κb signaling pathway. Biochem. Pharmacol..

[23] Li S., Lu J., Chen Y., Xiong N., Li L., Zhang J., Yang H., Wu C., Zeng H., Liu Y. (2017). Mcp-1-induced erk/gsk-3β/snail signaling facilitates the epithelial–mesenchymal transition and promotes the migration of mcf-7 human breast carcinoma cells. Cell. Mol. Immunol..

[24] Panee J. (2012). Monocyte chemoattractant protein 1 (mcp-1) in obesity and diabetes. Cytokine.

[25] Si M.-Y., Fan Z.-C., Li Y.-Z., Chang X.-L., Xie Q.-D., Jiao X.-Y. (2015). The prognostic significance of serum and cerebrospinal fluid mmp-9, ccl2 and svcam-1 in leukemia cns metastasis. J. Neuro-Oncol..

[26] Jiang Y.-J., Chao C.-C., Chang A.-C., Chen P.-C., Cheng F.-J., Liu J.-F., Liu P.-I., Huang C.-L., Guo J.-H., Huang W.-C. (2022). Cigarette smoke-promoted increases in osteopontin expression attract mesenchymal stem cell recruitment and facilitate lung cancer metastasis. J. Adv. Res..

[27] Achudhan D., Liu S., Lin Y., Lee H., Wang S., Huang W., Wu Y., Kuo Y., Tang C. (2022). Antcin K inhibits vegf-dependent angiogenesis in human rheumatoid arthritis synovial fibroblasts. J. Food Biochem..

[28] Lee H.-P., Chen P.-C., Wang S.-W., Fong Y.-C., Tsai C.-H., Tsai F.-J., Chung J.-G., Huang C.-Y., Yang J.-S., Hsu Y.-M. (2019). Plumbagin suppresses endothelial progenitor cell-related angiogenesis in vitro and in vivo. J. Funct. Foods.

[29] Lee H.-P., Wu Y.-C., Chen B.-C., Liu S.-C., Li T.-M., Huang W.-C., Hsu C.-J., Tang C.-H. (2020). soya-cerebroside reduces interleukin production in human rheumatoid arthritis synovial fibroblasts by inhibiting the erk, nf-κb and ap-1 signalling pathways. Food Agric. Immunol..

[30] Lee H.-P., Liu S.-C., Wang Y.-H., Chen B.-C., Chen H.-T., Li T.-M., Huang W.-C., Hsu C.-J., Wu Y.-C., Tang C.-H. (2021). Cordycerebroside a suppresses vcam-dependent monocyte adhesion in osteoarthritis synovial fibroblasts by inhibiting mek/erk/ap-1 signaling. J. Funct. Foods.

[31] Kadomoto S., Izumi K., Mizokami A. (2021). Roles of ccl2-ccr2 axis in the tumor microenvironment. Int. J. Mol. Sci..

[32] Iwamoto H., Izumi K., Mizokami A. (2020). Is the c-c motif ligand 2-c-c chemokine receptor 2 axis a promising target for cancer therapy and diagnosis?. Int. J. Mol. Sci..

[33] Dong Y., Zhang S., Zhao S., Xuan M., Zhu G., Zhang Z., Guo W. (2023). Ccl2 promotes lymphatic metastasis via activating rhoa and rac1 pathway and predict prognosis to some extent in tongue cancer. Cancer Biol. Ther..

[34] Zhang J., Lu Y., Pienta K.J. (2010). Multiple roles of chemokine (c-c motif) ligand 2 in promoting prostate cancer growth. J. Natl. Cancer Inst..

[35] Knopfová L., Biglieri E., Volodko N., Masařík M., Hermanová M., Garzón J.F.G., Dúcka M., Kučírková T., Souček K., Šmarda J. (2017). Transcription factor c-myb inhibits breast cancer lung metastasis by suppression of tumor cell seeding. Oncogene.

[36] Yoshimura T., Li C., Wang Y., Matsukawa A. (2023). The chemokine monocyte chemoattractant protein-1/ccl2 is a promoter of breast cancer metastasis. Cell Mol. Immunol..

[37] Sierra-Filardi E., Nieto C., Domínguez-Soto A., Barroso R., Sánchez-Mateos P., Puig-Kroger A., López-Bravo M., Joven J., Ardavín C., Rodríguez-Fernández J.L. (2014). Ccl2 shapes macrophage polarization by gm-csf and m-csf: Identification of ccl2/ccr2-dependent gene expression profile. J. Immunol..

[38] Lazennec G., Richmond A. (2010). Chemokines and chemokine receptors: New insights into cancer-related inflammation. Trends Mol. Med..

[39] Lamhamedi-Cherradi S.-E., Mohiuddin S., Mishra D.K., Krishnan S., Velasco A.R., Vetter A.M., Pence K., McCall D., Truong D.D., Cuglievan B. (2021). Transcriptional activators yap/taz and axl orchestrate dedifferentiation, cell fate, and metastasis in human osteosarcoma. Cancer Gene Ther..

[40] Zhang S., Qin Y.P., Kuang J.M., Liu Y.H. (2017). Proteomic investigation of resistance to chemotherapy drugs in osteosarcoma. Technol. Health Care Off. J. Eur. Soc. Eng. Med..

[41] Biazzo A., De Paolis M. (2016). Multidisciplinary approach to osteosarcoma. Acta Orthop. Belg..

[42] Bajpai M., Pardhe N., Chandolia B., Arora M. (2017). Osteogenic sarcoma of mandible. J. Coll. Physicians Surg. Pak. JCPSP.

[43] Gebhard C., Miller I., Hummel K., Ondrovics M.N.N., Schlosser S., Walter I. (2018). Comparative proteome analysis of monolayer and spheroid culture of canine osteosarcoma cells. J. Proteom..

[44] Bacci G., Rocca M., Salone M., Balladelli A., Ferrari S., Palmerini E., Forni C., Briccoli A. (2008). High grade osteosarcoma of the extremities with lung metastases at presentation: Treatment with neoadjuvant chemotherapy and simultaneous resection of primary and metastatic lesions. J. Surg. Oncol..

[45] Chao C.-C., Lee W.-F., Yang W.-H., Lin C.-Y., Han C.-K., Huang Y.-L., Fong Y.-C., Wu M.-H., Lee I.-T., Tsai Y.-H. (2021). Igfbp-3 stimulates human osteosarcoma cell migration by upregulating vcam-1 expression. Life Sci..

[46] Lee C.-W., Chiang Y.-C., Yu P.-A., Peng K.-T., Chi M.-C., Lee M.-H., Fang M.-L., Lee K.-H., Hsu L.-F., Liu J.-F. (2021). A role of cxcl1 drives osteosarcoma lung metastasis via vcam-1 production. Front. Oncol..

[47] Wang S.-W., Wu H.-H., Liu S.-C., Wang P.-C., Ou W.-C., Chou W.-Y., Shen Y.-S., Tang C.-H. (2012). Ccl5 and ccr5 interaction promotes cell motility in human osteosarcoma. PLoS ONE.

[48] Levinson H., Hopper J.E., Ehrlich H.P. (2002). Overexpression of integrin alphav promotes human osteosarcoma cell populated collagen lattice contraction and cell migration. J. Cell. Physiol..

[49] Tsai H.-C., Lai Y.-Y., Hsu H.-C., Fong Y.-C., Lien M.-Y., Tang C.-H. (2021). Ccl4 stimulates cell migration in human osteosarcoma via the mir-3927-3p/integrin αvβ3 axis. Int. J. Mol. Sci..

[50] Wu C.-L., Tsai H.-C., Chen Z.-W., Wu C.-M., Li T.-M., Fong Y.-C., Tang C.-H. (2013). Ras activation mediates wisp-1-induced increases in cell motility and matrix metalloproteinase expression in human osteosarcoma. Cell. Signal..

[51] Yang M.-D., Lin K.-C., Lu M.-C., Jeng L.-B., Hsiao C.-L., Yueh T.-C., Fu C.-K., Li H.-T., Yen S.-T., Lin C.-W. (2017). Contribution of matrix metalloproteinases-1 genotypes to gastric cancer susceptibility in Taiwan. BioMedicine.

[52] Puppo M., Valluru M.K., Clézardin P. (2021). Micrornas and their roles in breast cancer bone metastasis. Curr. Osteoporos. Rep..

[53] Shen D., Zhao H., Gu A., Wu Y., Weng Y., Li S., Song J., Gu X., Qiu J., Zhao W. (2021). Mirna-10a-5p inhibits cell metastasis in hepatocellular carcinoma via targeting ska1. Kaohsiung J. Med. Sci..

[54] Tang J., Chen J., Wang Y., Zhou S. (2022). The role of mirna-433 in malignant tumors of digestive tract as tumor suppressor. Cancer Rep..

[55] Peng Y., Croce C.M. (2016). The role of micrornas in human cancer. Signal Transduct. Target. Ther..

[56] Azimi M., Totonchi M., Ebrahimi M. (2022). Determining the role of micrornas in self-renewal, metastasis and resistance to drugs in human gastric cancer based on data mining approaches: A systematic review. Cell J..

